# Monitoring chest compression rate in automated external defibrillators using the autocorrelation of the transthoracic impedance

**DOI:** 10.1371/journal.pone.0239950

**Published:** 2020-09-30

**Authors:** Sofía Ruiz de Gauna, Jesus María Ruiz, Jose Julio Gutiérrez, Digna María González-Otero, Daniel Alonso, Carlos Corcuera, Juan Francisco Urtusagasti

**Affiliations:** 1 Department of Communications Engineering, University of the Basque Country, UPV/EHU, Bilbao, Spain; 2 Bexen Cardio, Ermua, Spain; 3 Emergentziak-Osakidetza, The Basque Country Health System, the Basque Country, Spain; University of Palermo, ITALY

## Abstract

**Aim:**

High-quality chest compressions is challenging for bystanders and first responders to out-of-hospital cardiac arrest (OHCA). Long compression pauses and compression rates higher than recommended are common and detrimental to survival. Our aim was to design a simple and low computational cost algorithm for feedback on compression rate using the transthoracic impedance (TI) acquired by automated external defibrillators (AEDs).

**Methods:**

ECG and TI signals from AED recordings of 242 OHCA patients treated by basic life support (BLS) ambulances were retrospectively analyzed. Beginning and end of chest compression series and each individual compression were annotated. The algorithm computed a biased estimate of the autocorrelation of the TI signal in consecutive non-overlapping 2-s analysis windows to detect the presence of chest compressions and estimate compression rate.

**Results:**

A total of 237 episodes were included in the study, with a median (IQR) duration of 10 (6–16) min. The algorithm performed with a global sensitivity in the detection of chest compressions of 98.7%, positive predictive value of 98.7%, specificity of 97.1%, and negative predictive value of 97.1% (validation subset including 207 episodes). The unsigned error in the estimation of compression rate was 1.7 (1.3–2.9) compressions per minute.

**Conclusion:**

Our algorithm is accurate and robust for real-time guidance on chest compression rate using AEDs. The algorithm is simple and easy to implement with minimal software modifications. Deployment of AEDs with this capability could potentially contribute to enhancing the quality of chest compressions in the first minutes from collapse.

## Introduction

Early, high-quality cardiopulmonary resuscitation (CPR) is a key contributor to maximizing survival from out-of-hospital cardiac arrest (OHCA). The immediate initiation of CPR can double or quadruple survival [1–3]. Current resuscitation guidelines indicate compression targets for rate, depth, chest recoil, and chest compression fraction [4, 5]. However, providing chest compressions in adherence to recommendations is generally difficult in the field even for well-trained rescuers [6, 7]. The most frequent non-compliances are long pauses in chest compressions and high compression rates resulting in low compression depths, all these decreasing the likelihood of restoration of spontaneous circulation and survival [7–11].

Monitoring the compression technique to provide real-time guidance to rescuers contributes to improving CPR quality [12]. Principally used by the advanced life support (ALS), CPR feedback usually relies on advanced systems based on accelerometers, force sensors or magnetic induction which can be connected to the monitor-defibrillator or used as stand-alone devices [13]. Unfortunately, these advanced systems are not generally available for basic life support (BLS) bystanders and first responders using automated external defibrillators (AEDs).

Most current AEDs acquire the transthoracic impedance (TI) signal together with the ECG through defibrillation pads. TI represents the resistance of the thorax to electrical current flow. It is used to check for proper electrode contact and adjust the defibrillation energy. Oscillations in the TI signal are correlated with ventilations, chest compressions or presence of circulation [14]. In particular, chest compression activity can be observed in the TI signal in the form of fluctuations around the patient’s baseline impedance synchronized with each compression [15]. Methods for the automated detection of chest compressions have been proposed using several TI features derived from the time domain [16], the frequency domain [17] and the combination of both [18, 19]. In fact, some defibrillators implement proprietary algorithms to provide CPR feedback based on the TI. These methods were generally tested with signal segments extracted from OHCA episodes collected by the ALS using monitor-defibrillators with CPR feedback.

In this context, we wanted to design a simple algorithm for detecting compression activity and computing compression rate in real-time that could be used in BLS settings. The algorithm had to be robust to account for the large dispersion in compression rates observed in the field. Additionally, it had to be simple enough to be implemented in low-cost AEDs with low computational power. We hypothesized that the autocorrelation of the TI signal could provide the necessary information for that purpose. The method was optimized and evaluated retrospectively with AED recordings from OHCA treated by a BLS ambulance service.

## Materials and methods

### Data collection

Data for the study came from the BLS ambulance system of Emergentziak-Osakidetza, the emergency medical services system in the Basque Country (Spain). Emergentziak-Osakidetza serves a population of approximately 2 200 000 inhabitants, with a density of about 300 inhabitants per km^2^ (4 429 per km^2^ in the urban area). We gained access to all LIFEPAK^®^ 1000 (Stryker, formerly Physio-Control, USA) AED recordings collected from consecutive patients from 2013 through 2014 that were available in the regional emergency services central office. Recordings belonged to the out-of-hospital cardiac arrest registry (OHSCAR). The regional prospective collection was approved by the Ethical Committee of Clinical Research of the Basque Country (CEIC-E). No patient private information was included in the database.

LIFEPAK 1000 AEDs stored ECG and TI signals in their internal memory. Prior to their storage, the ECG was band-pass filtered to suppress direct current and high frequency noise, and the TI was high-pass filtered to suppress patient’s baseline impedance. We extracted ECG and AED signals from the AED recordings and exported them to Matlab^®^ (Mathworks, USA) format. Then, signals were resampled to a common sampling frequency of 250 Hz.

### Data annotation

Signal intervals corresponding to shock administration, disconnections of defibrillation pads and file storage errors were discarded from further analysis. ECG and TI signals were reviewed to annotate the beginning and end of each chest compression interval within the episodes. Each individual chest compression was identified by its corresponding fluctuation in the TI signal. We developed a custom-made software with a graphical user interface for annotation tasks. Experts with large experience in analyzing AED signals were in charge of the annotation process. They jointly reviewed and annotated a subset of the episodes in order to define the annotation rules. The remaining episodes were randomly split in three parts, and each part was annotated by a single reviewer.

**Fig 1:**
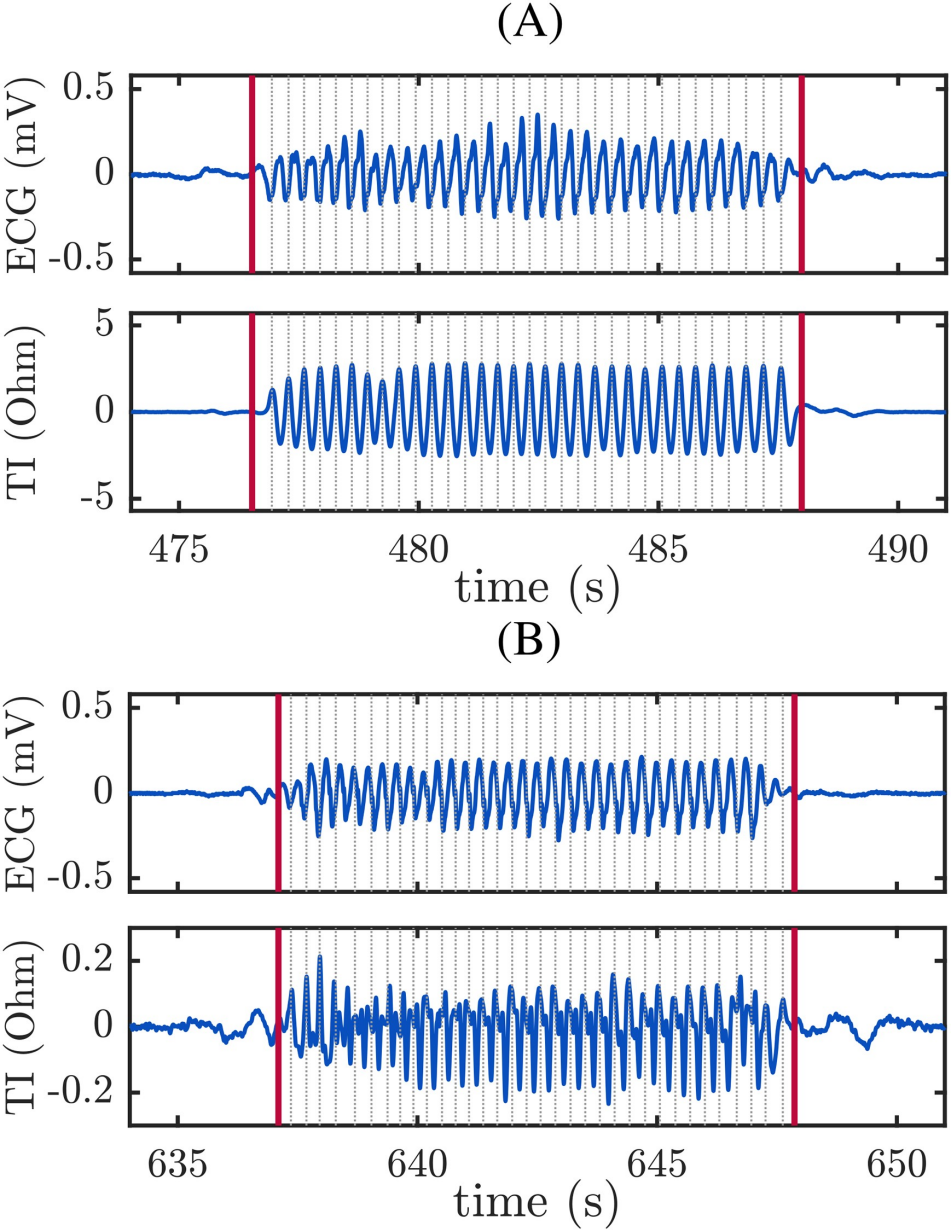
Examples of compression intervals identified in the AED signals. Intervals are delimited with vertical red lines. In both examples, oscillations caused by chest compressions are visible in the ECG (top) and the TI (bottom). Individual chest compressions are marked with grey dashed lines.

The quality of the TI signal was generally sufficient for the reliable identification of individual chest compressions, but in some cases the ECG was used for confirmation. Fig 1 shows two signal segments, with chest compression series delimited by red lines. Each individual compression is depicted with a grey dotted line. In example (A), compressions are clearly distinguishable both in the ECG and in the TI. In example (B), however, compressions are more easily identifiable in the ECG.

### Algorithm description

We developed an algorithm to estimate compression rates in the range 60–250 cpm. This way, the algorithm could detect and correct severe rate disagreements with respect to current guidelines recommendation. For that purpose, it processes the TI signal in consecutive non-overlapped 2-s analysis windows. The window size was chosen to ensure that a window comprised at least two compressions even at the slowest rate to be detected (60 cpm). The algorithm was designed to provide an estimate of the chest compression rate every 2 s if compression activity is detected, or a zero value, otherwise.

The first step involves low-pass filtering the 2-s TI signal interval to enhance the fundamental component of the fluctuation caused by chest compressions. Then, the algorithm computes a biased estimate of the autocorrelation of the filtered TI. This provides a measurement of the similarity of the signal with its delayed version. When the TI waveform shows regular fluctuations, the autocorrelation presents a maximum at the time-lag corresponding to one period of the fluctuation. The position of the maximum represents the average time between consecutive chest compressions. Its inverse is an estimate of the average compression rate in the analyzed 2-s window. We designed the algorithm to locate a peak exceeding a predefined threshold in the lag range from 0.24 to 1.0 s. This corresponds to the range of compression rates to be detected (60–250 cpm).

During a chest compression pause, the autocorrelation of the TI will have a disorganized waveform not presenting a prominent maximum. If there is no peak satisfying the amplitude condition in the fixed lag range, the output of the algorithm for compression rate is zero, i.e. the analyzed window is classified as “no chest compressions”.

Further details on the algorithm and some graphical examples are provided in the supplementary materials (S1 Appendix).

### Performance evaluation

Episodes were randomly split into training (15% of the recordings) and validation subsets. The algorithm was designed and optimized with the training subset and evaluated with the validation subset. We launched the algorithm from the beginning to the end of the TI signal available per episode. This way we evaluated the algorithm as closest as possible to its real performance when implemented in an AED.

First, the method was evaluated in terms of its ability to detect compression activity. All the outputs of the algorithm (one value every 2 s) were classified in one of the following four categories:

- TP (true positive): the algorithm reported a non-zero compression rate value for a window with annotated chest compressions.
- TN (true negative): the algorithm reported a zero value for a window with no chest compressions.
- FP (false positive): the algorithm reported a non-zero compression rate value for an analysis window with no chest compressions.
- FN (false negative): the algorithm reported a zero value for a window with annotated chest compressions.

Then, according to this categorization, the algorithm was evaluated in terms of its ability to detect chest compression activity through the following figures of merit:

- Sensitivity (Se): percentage of annotated compression windows that were correctly detected (i.e. $100\cdot \frac{TP}{TP+FN}$).
- Positive predictive value (PPV): percentage of detected compression windows that actually contained compressions (i.e. $100\cdot \frac{TP}{TP+FP}$).
- Specificity (Sp): percentage of annotated no-compression windows that were correctly detected (i.e. $100\cdot \frac{TN}{TN+FP}$).
- Negative predictive value (NPV): percentage of detected no-compression windows that actually did not contain compressions (i.e. $100\cdot \frac{TN}{TN+FN}$).

These figures of merit were computed both globally, that is, by jointly analyzing all the 2-s windows in all the episodes of the validation subset, and per episode, that is, for the 2-s windows comprised by each episode separately.

Second, the method was evaluated in terms of its ability to reliably estimate compression rate. The reference for compression rate was computed from the annotations as the inverse of the mean time between the compression instances manually annotated in the 2-s analysis window (see Data annotation). Error in compression rate estimation was computed as the difference between the reference values and the algorithm estimates.

Third, the method was evaluated in terms of its ability to reliably estimate chest compression fraction (CCF). The reference CCF per episode was computed as the percentage of 2-s windows with annotated chest compressions. Error in CCF estimation was computed as the difference between the reference value and the algorithm estimate.

### Statistical analysis

Distributions were reported as median (IQR) as they did not pass Lilliefors normality test. Global and per episode figures of merit with their 95% confidence intervals (CI) were computed.

## Results

From a database of 242 AED recordings (one per patient) containing continuous and concurrent ECG and TI signals, 237 were included in the study. Two episodes were discarded because the ECG was compatible with spontaneous circulation and no chest compressions were observed. Three episodes were discarded because the signals were corrupted by noise impeding reliable annotation of chest compression instances. The training set contained 30 episodes and the validation set 207 episodes.

**Fig 2:**
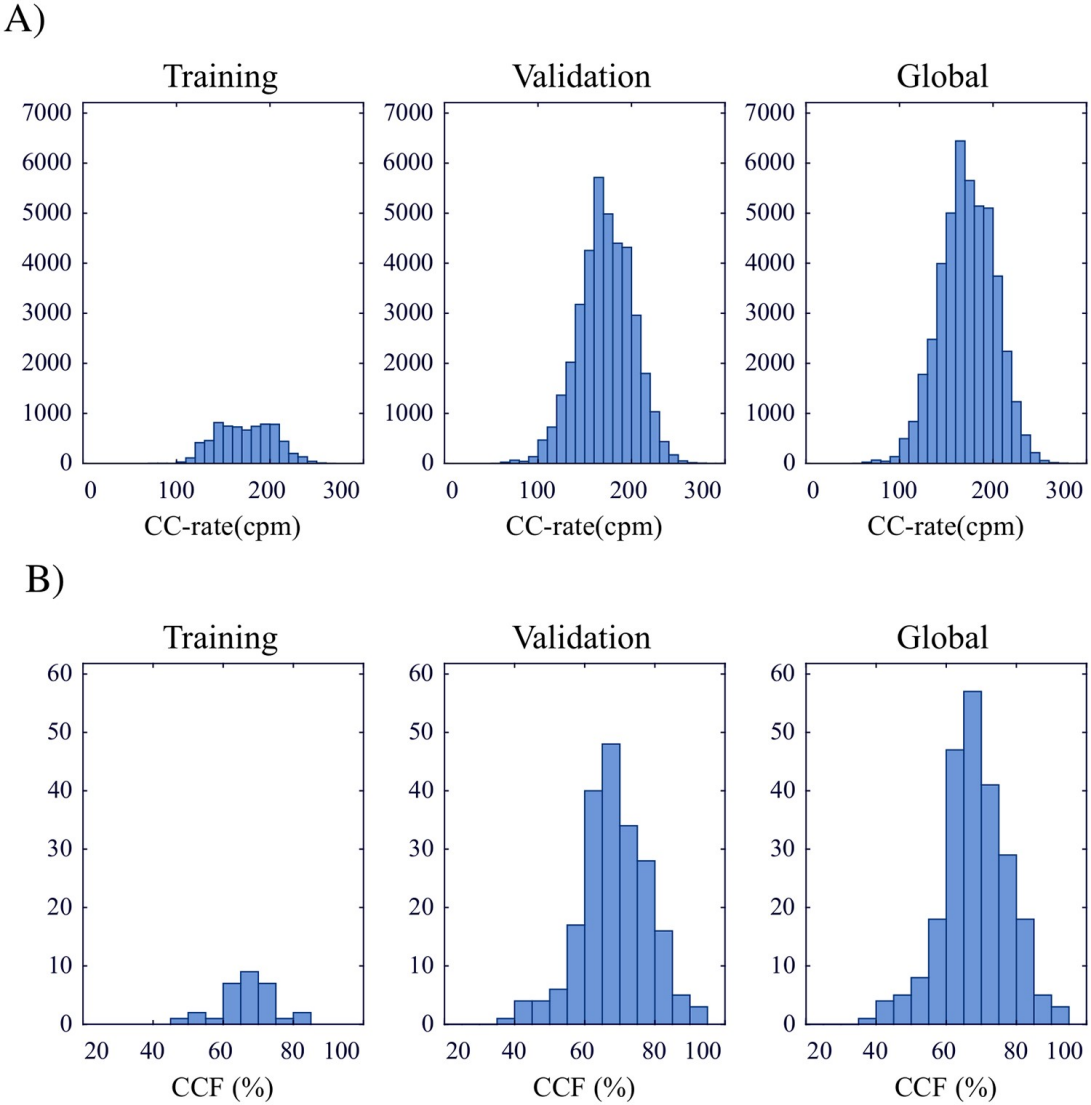
Distributions of chest compression rate (CC-rate) (A) and CCF (B) in the training and validation subsets and global distributions (both subsets jointly). Values were computed from the reference standard annotations.

A total of 2766.6 min were reviewed and annotated: 449.7 min in the training subset and 2316.9 min in the validation subset. A total of 303,548 chest compressions were annotated: 47,937 (training) and 255,611 (validation). Finally, a total of 66,610 analysis windows were processed by the algorithm: 10,682 (training) and 55,478 (validation). Table 1 summarizes the characteristics of the episodes included in the study. Fig 2 shows the distributions of compression rate (A) and CCF (B) in the AED database. Distributions are reported separately for the training and validation subsets and also jointly (global distributions).

**Table 1:** Characteristics of the database (derived from the annotations).

	Training	Validation	Total
**Episodes (n)**	30	207	237
**Duration (min)**	12.9 (6.3–21.1)	9.5 (5.7–14.8)	10.0 (5.9–16.1)
**Compressions (n)**	1 406 (604–2 005)	1 055 (640–1 667)	1 073 (637–1 802)
**2-s windows (n)**	285 (158–498)	217 (140–349)	233 (142–372)
**mean compression rate (cpm)**	170 (154–197)	173 (158–189)	173 (157–190)
**compression rate range (cpm)**	77.3–263.2	56.4–294.1	56.4–294.1
**CCF (%)**	66.9 (62.5–72.4)	68.3 (62.5–75.0)	67.7 (62.5–74.2)

Values are reported per episode except for compression rate range, which was computed from all annotated 2-s windows. cpm: compressions per minute. CCF: chest compression fraction.

**Fig 3:**
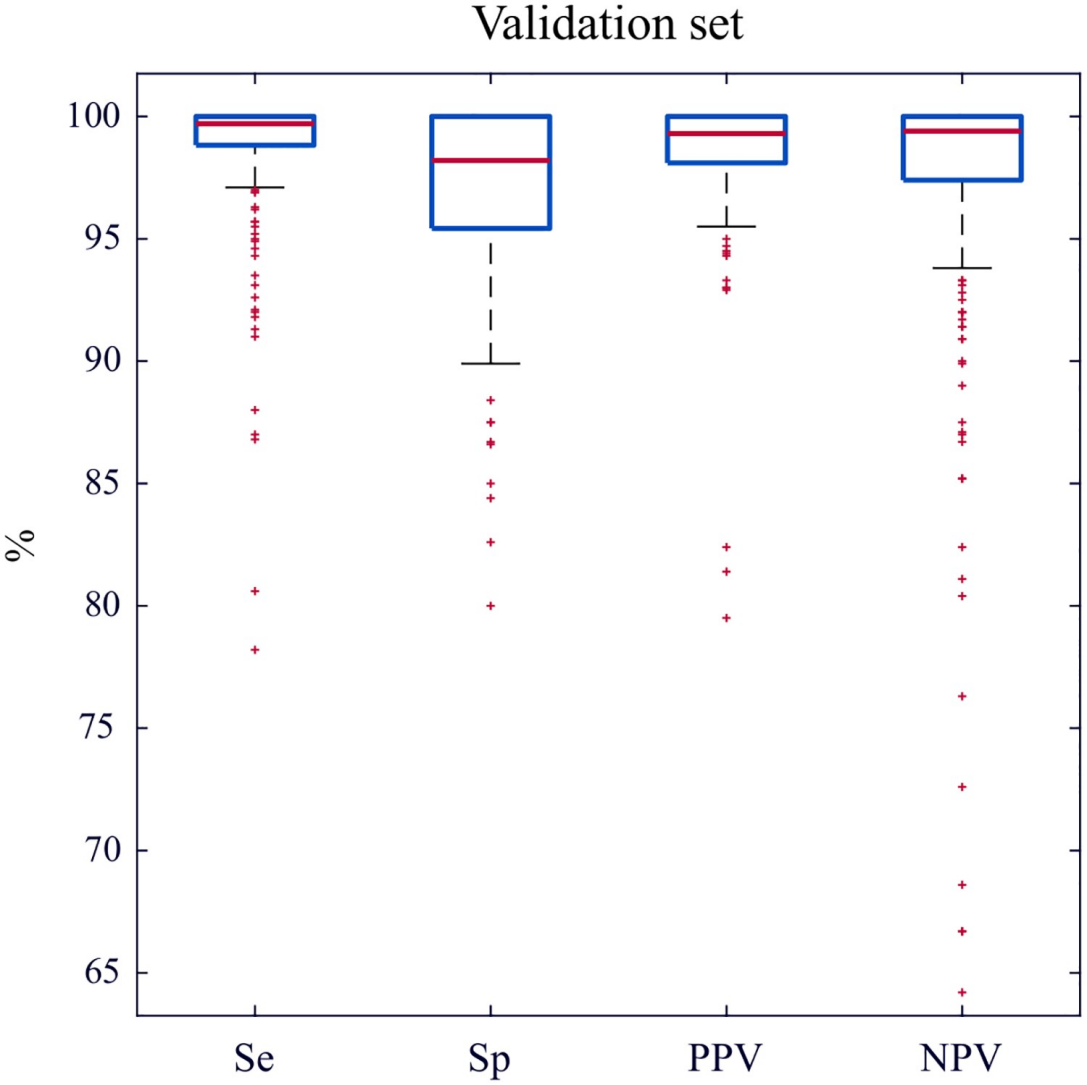
Algorithm performance metrics. Distributions per episode in the validation subset.

Considering independence between the diagnoses of the 55,478 2-s windows in the validation subset, global algorithm performance was: Se/PPV 98.7%/98.7%, Sp/NPV 97.1%/97.1%. In the per episode analysis, mean performance values and their CI were: Se 98.6% (95% CI: 98.2–99.1), PPV 98.5% (95% CI: 98.1–98.9), Sp 97.1% (95% CI: 96.6–97.6), and NPV 96.9% (95% CI: 96.1–97.8). Fig 3 shows the distributions per episode.

**Fig 4:**
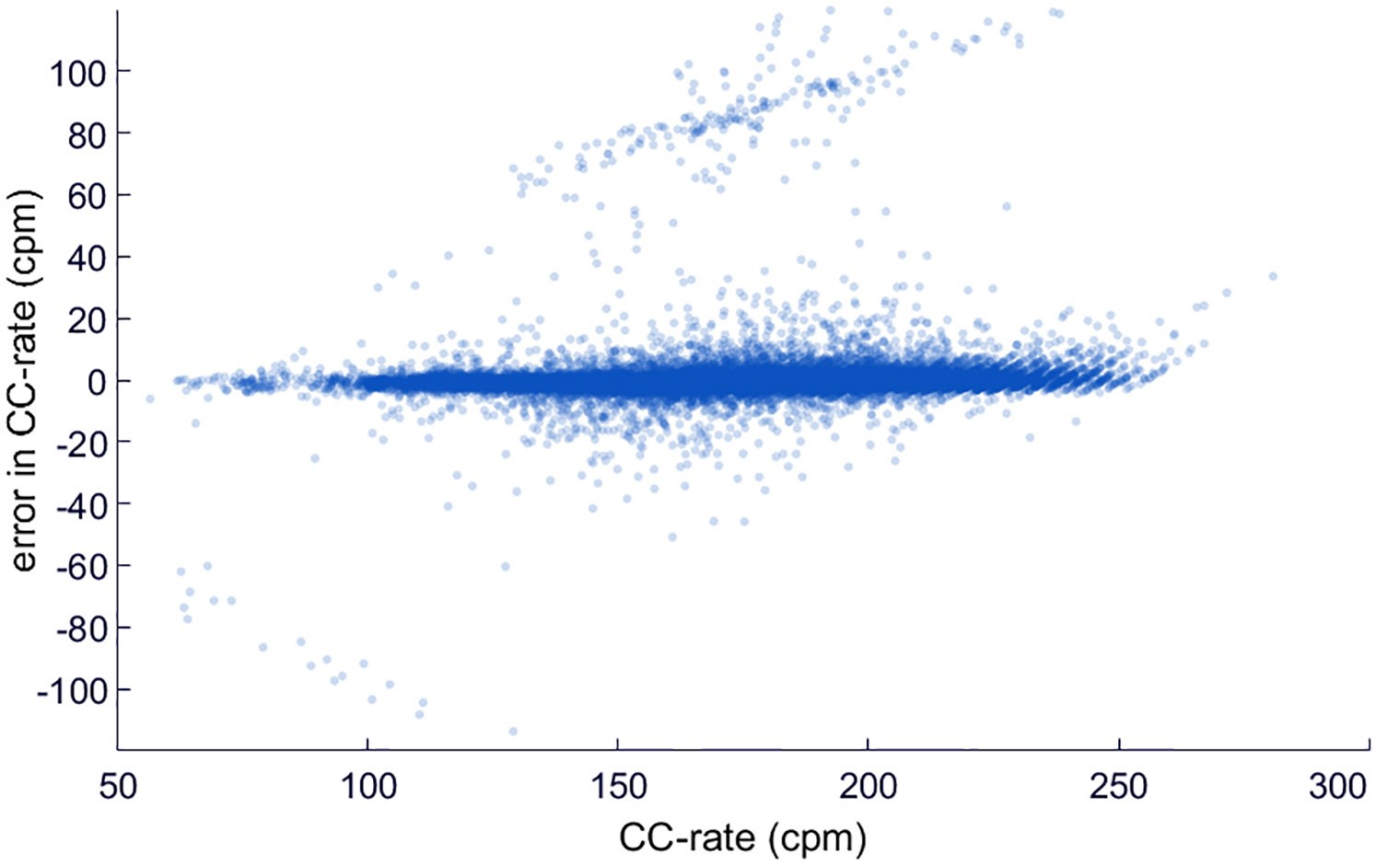
Error in the estimation of compression rate. Scatter plot representing the error in cpm with respect to the reference value for the validation subset.

The median unsigned error in the estimation of the chest compression rate per episode was 1.7 (1.3–2.9) cpm. Fig 4 depicts the error in the estimated rate as a function of the reference. The error was below ±10 cpm in 97.7% of the analyzed windows containing chest compressions.

**Fig 5:**
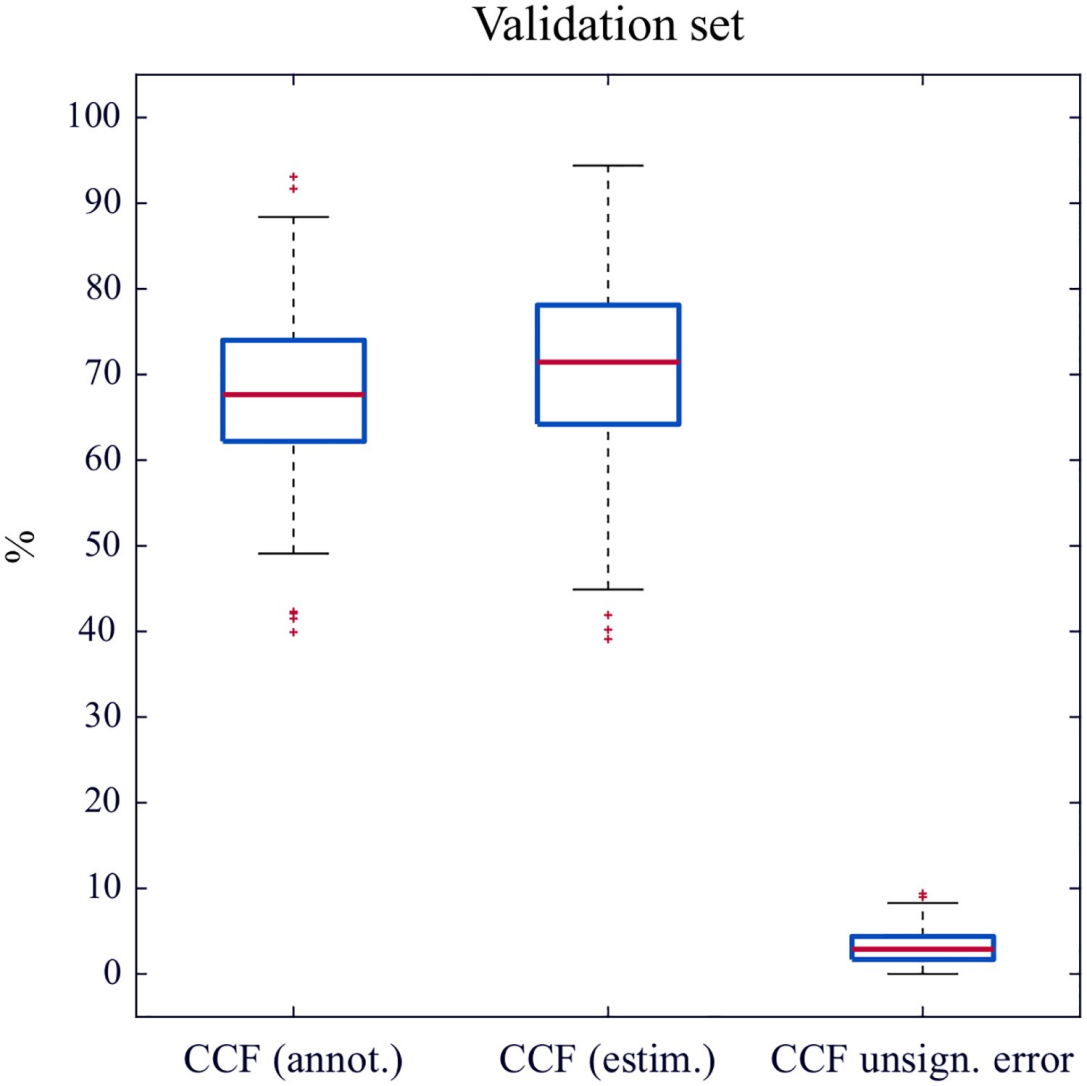
Computation of CCF in the validation subset. From left to right, distributions of the CCF computed from the annotations, estimated by the algorithm, and unsigned error in the estimation.

The unsigned error in the estimation of CCF was 2.9% (1.7–4.4). Fig 5 shows the distributions of CCF per episode calculated from the reference and by the algorithm, and the distribution of the unsigned error.

## Discussion

The use of monitoring and feedback devices that guide CPR quality during resuscitation contributes to meeting quality recommendations [12]. These devices are mostly used in ALS settings, since most monitor-defibrillators already incorporate advanced technology for measuring chest compression quality (generally based on accelerometers or on electromagnetic fields). However, this is still a challenge in BLS settings, as many AEDs do not incorporate any type of CPR feedback. Fitting all AEDs (even low-cost devices) with CPR feedback capabilities to guide rescuers on compression performance could be a significant step forward to achieve the goal of early, high-quality CPR provided by bystanders and first responders.

Minimizing interruptions in chest compressions and providing adequate compression rates are two critical CPR quality components [4, 5, 12]. Interruptions in chest compressions compromise blood flow to the heart and brain and decrease defibrillation success and survival [20, 21]. Guidelines recommend a tight regulation of compression rate to the target range of 100–120 cpm; at lower rates adequate forward flow may not be generated, while too high rates may decrease compression depth and impede proper heart refilling, reducing the effectiveness of chest compressions [9].

In this context, we proposed an algorithm to detect chest compression activity and estimate compression rate to be used for feedback in BLS settings. It is based solely on the TI signal available in most commercial AEDs. The algorithm can operate in real-time computing a value for compression rate every 2-s that could be displayed to the rescuer during the resuscitation attempt with convenient timing to provide adequate guidance. It can also be launched after the event for debriefing purposes.

The algorithm was accurate in the detection of compression activity, reporting Se and PPV above 98% and Sp and NPV above 97%. These results are comparable to those reported by previous methods relying on the TI signal with more complex approaches [16–19]. Alonso et al. reported a Se/PPV of 97%/97% using the amplitudes and durations of each TI oscillation (time-domain approach) [16]. González-Otero et al. reported a Se/PPV of 96%/97% with 2-s analysis windows and TI features computed in the frequency domain [17]. Kwok et al. reduced the analysis window to 1-s, and used TI features computed in both the time and frequency domains, and a Hidden Markov model to improve accuracy [18]. Reported Se and PPV were 99% and 98%, respectively. More recently, Coult et al. combined TI features from the time and frequency domain computed in 5-s TI segments and reported Se and Sp above 98% [19]. We have demonstrated that the information conveyed in the TI autocorrelation was equally reliable with a simpler approach. Furthermore, all the cited studies used OHCA recordings collected by the ALS using monitor-defibrillators. We used BLS AED recordings since our method was intended to be applied in that setting.

Accurate online detection of presence/absence of compression activity is relevant for several reasons, particularly in BLS settings with lay people in the field: first, alerting rescuers on long pauses in chest compressions could contribute to reducing no-flow time. Second, the ability to detect programmed compression pauses (for example, pauses for ventilation in the 30:2 protocol or pauses during rescuers switch) could be used to launch automated analyses of the ECG. According to current guidelines, CPR has to be interrupted every 2 min for rhythm analysis, as chest compressions induce artifact in the ECG that could lead to an incorrect diagnosis by the AED algorithm.

Some manufacturers have tried to avoid these interruptions by designing a shock advise algorithm that can diagnose the corrupted ECG during chest compressions. Our method has the advantage of using the standard shock advice algorithm during chest compression pauses, which could reduce the implementation challenge and the time to market [22]. Furthermore, AEDs could have the capability of detecting circulation through the automated analysis of the ECG and the TI recorded during pauses for ventilation, as proposed in reference [23]. In this reference, the presence of QRS complexes in the ECG and a circulation component in the TI during the pause are used for automated circulation assessment. For that purpose, a robust and accurate method like the one we describe in the present study is pivotal.

Our algorithm was also accurate in the estimation of chest compression rate, and could help rescuers to adhere to the recommended 100–120 cpm range during resuscitation. We reported a median error of 1.7 cpm, comparable to other studies (1.2 cpm [16], 1.8 cpm [17], and 1.8 cpm [18]). Our algorithm was designed to detect chest compression rates between 60 and 250 cpm, a range much wider than current recommendations. Thus, the algorithm could be useful to bystanders with no experience in cardiopulmonary resuscitation.

Finally, the algorithm could be a powerful tool for the retrospective analysis of CPR quality in debriefing sessions. Post-event assessment of CCF, mean and instantaneous compression rate, location and duration of the compression pauses are key in reporting the quality of CPR [24].

The main advantages of the proposed algorithm are its high accuracy in the detection of the presence of chest compressions and in the estimation of compression rate. The algorithm relies on a single feature computed from the TI autocorrelation, i.e., the method is simple and of low computational cost. Consequently, the algorithm could be incorporated in commercial AEDs with minimal software modifications. Finally, the algorithm is robust, since it reliably performs in a wide range of compression rate conditions. However, integrating the method in AEDs and evaluating how well it performs in a BLS system would require further investigation.

### Limitations

Data used in the study came from a single AED model, and results may be dependent on the specific acquisition characteristics of the defibrillator. Generalizability of the results would benefit if the algorithm were tested with a larger database. Another limitation is that compressions were manually annotated in the TI signal used for designing the method. Therefore the algorithm replicated the annotations made by the experts. However, no other references such as acceleration or force signals are usually available in AED recordings.

Compression rates in our dataset were much higher than guidelines recommendations and showed a high dispersion. This favored the design of a robust algorithm but it is unusual to find BLS recordings with such high rates. When some of the BLS crew members were questioned about the matter, they admitted their tendency to compress the chest too fast at the time the study database was collected. They recognized the importance of training, and the use of metronome and feedback systems in the field. In the past years, CPR quality training programs have been developed in the Basque Country BLS system.

## Conclusion

Monitoring of chest compression activity and compression rate based on the autocorrelation of the TI signal is reliable. Our algorithm is simple and easy to implement in current AEDs to guide rescuers during CPR and could also be used for debriefing purposes. Since the TI signal is routinely acquired by most AEDs through defibrillation pads, incorporating this functionality in AEDs could potentially improve the quality of chest compressions provided by bystanders and first responders.

## Supporting information

S1 Appendix Description of the algorithm. Technical details of the designed algorithm with graphical examples.
(PDF)

S1 File Algorithm results. Main values for the algorithm operation and the corresponding reference values for all the analyzed windows in the validation subset.
(XLSX)
