# Supplementary material for: Monitoring chest compression rate in automated external defibrillators using the autocorrelation of the transthoracic impedance
Source: PLoS One. 2020 Sep 30;15(9):e0239950. doi: 10.1371/journal.pone.0239950 (PMC7526915; doi:10.1371/journal.pone.0239950)
Supplement: S1 Appendix — Technical details of the designed algorithm with graphical examples. (PDF) [file pone.0239950.s001.pdf]

Appendix. Estimation of chest compression rate using the autocorrelation of the transthoracic impedance signal

LIFEPAK 1000 AEDs stored ECG and TI signals in their internal memory. Prior to their storage, the ECG was band-pass filtered to suppress direct current and high frequency noise, and the TI was high-pass filtered to suppress patient's baseline impedance. We extracted ECG and TI signals from the AED recordings and exported them to Matlab (Mathworks, USA) format. Then, we resampled the signals to a common sampling frequency of 250 Hz.

Description of the algorithm

The algorithm processes the transthoracic impedance (TI) signal in consecutive non-overlapped 2-s analysis windows. First, the samples of the raw TI signal are low-pass filtered to enhance the fundamental component of the fluctuation caused by chest compressions. The low-pass filter parameters were: Butterworth approximation, 5th order, cut-off frequency 4Hz. Then, the algorithm computes a biased estimate of the autocorrelation of the filtered TI and searches for a peak in the autocorrelation above a defined amplitude threshold in the lag range from 0.24 to 1.0 s. This implies that the algorithm is able to detect compression rates from 60 to 250 cpm.

For the autocorrelation to give us information about compression rate, we needed a time window long enough to comprise several compressions in the range of rates we wanted to detect, but short enough to obtain a "continuous feedback". We wanted an algorithm capable of detecting compression rates in a range much wider than the recommended rates, in order to be able to provide useful feedback to rescuers using automated external defibrillators without metronomes or in a very noisy environment. In addition, we wanted the algorithm to be useful to bystanders with no experience in cardiopulmonary resuscitation providing very slow chest compressions because of fear or fatigue. The low range we used in the algorithm design was 60 compressions per minute (1 compression per second). Therefore, the chosen window size to have at least two compressions at this lowest rate was 2 s.

The position of the peak in the autocorrelation, T, represents the averaged time period between chest compressions in the analyzed window. Consequently, chest compression rate, r_{cc}, can be computed using Eq (1):

$$r_{cc} = 60 \cdot \frac{1}{T} \text{ (cpm)} \tag{1}$$

If there is no peak satisfying the amplitude condition in the fixed lag range, the algorithm output for compression rate is $r_{cc} = 0$ cpm, i.e. the analyzed windows is classified as "no chest compressions".

Fig 1 shows an example of the operation of the algorithm. The first two panels show the raw and the filtered TI signal, respectively. The low pass filter enhances the fundamental component of the chest compression fluctuation, so that the TI presents a sinusoidal waveform. The low panels in the figure depict the operation of the algorithm in the absence (left) and presence (right) of chest compressions. The processed 2-s window is depicted by two red dotted vertical lines. In the first example, no chest compressions are present: no significant peak above the fixed threshold, th, is detected in the TI autocorrelation. The output of the algorithm is $r_{cc} = 0$ cpm. In the second example, there is a prominent peak successfully detected in the 0.24 and 1.0 s lag range. Its position in seconds, T, allows the estimation of compression rate using Eq (1).

Graphical examples

Fig 2 illustrates some of the few cases of algorithm misclassification. Although with a low incidence in our experiments, these errors have different implications during the course of resuscitation or in post-event debriefing sessions. Examples A and B correspond to false positive (FP) events. In example A, the regular fluctuations in the TI caused a peak in the autocorrelation which was detected by the algorithm. This small acceleration activity was probably caused by rescuer's accidental leaning on the patient's chest. In example B, the larger fluctuations can be attributable to patient's movement or disturbances in the skin-electrode contact. These errors have little effect during the intervention since rescuers would ignore feedback, but could cause overestimation of chest compression fraction (CCF).

Examples C and D correspond to false negative (FN) events. In example C, TI amplitude decreases gradually during chest compressions (while the amplitude of the compression artifact in the ECG keeps stable). At the end of the compression series, TI waveform presents low amplitude and lack of regularity. The algorithm failed to provide feedback on compression rate for that 2-s interval. Example D shows a FN event in the middle of the compression series, caused by the low TI amplitude in the analyzed window. Implications of FN events are twofold: rescuers would receive no feedback during these intervals and CCF could be underestimated. These errors were scarce and isolated in our dataset (i.e. non-occurring in consecutive 2-s intervals), causing a minimal impact on rescuer's action.

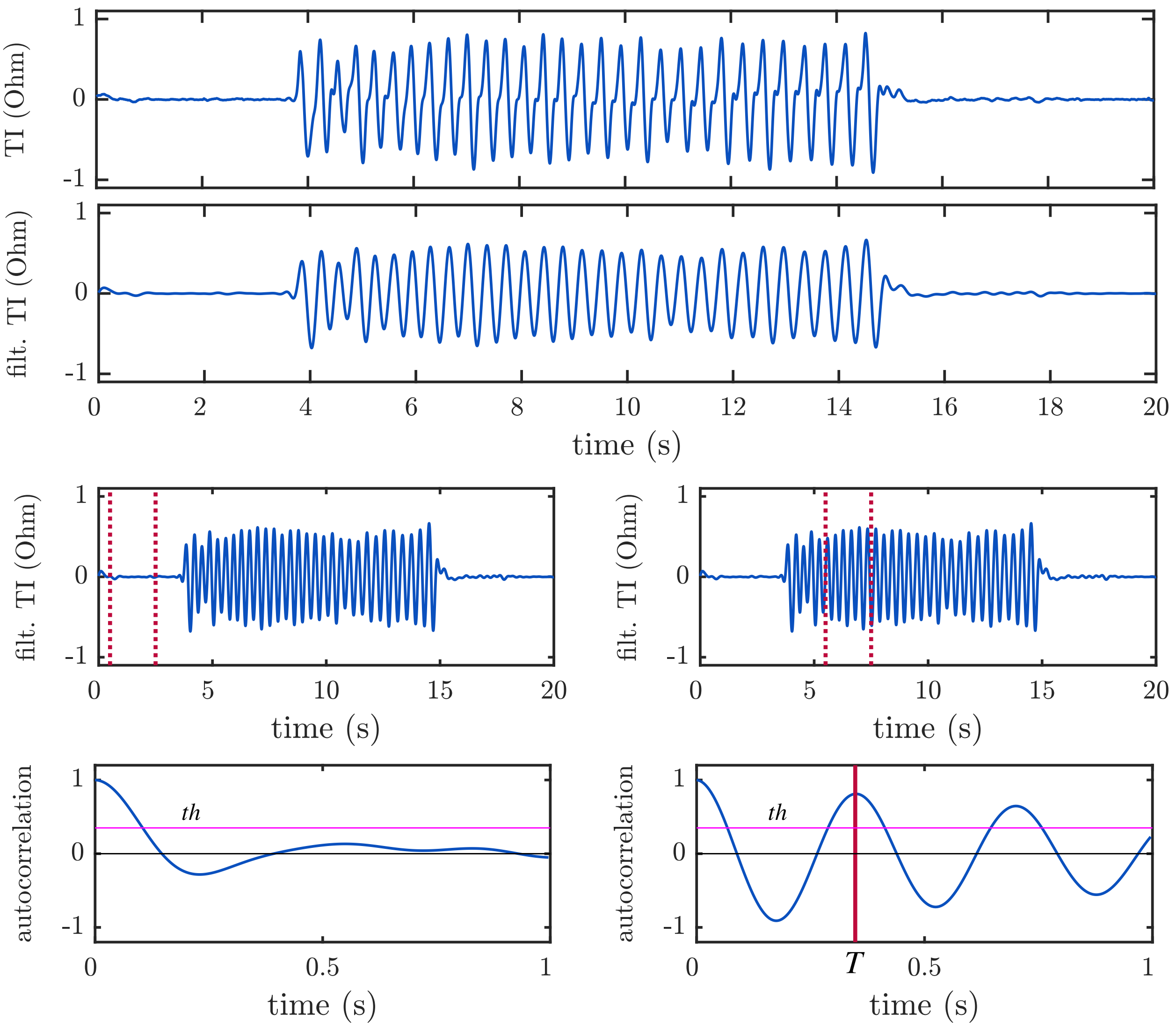

Fig 1. Graphical description of the algorithm operation. First panel: original TI signal; Second panel: low-pass filtered TI; Third and four panels, left: 2-s analysis window during no compressions, depicted with red dotted lines; the autocorrelation shows no peak above the threshold, th, therefore $r_{cc} = 0\,\text{cpm}$. Third and four panels, right: 2-s analysis window during compressions; the peak in the autocorrelation yields an estimate of r_{cc}, according to Eq (1).

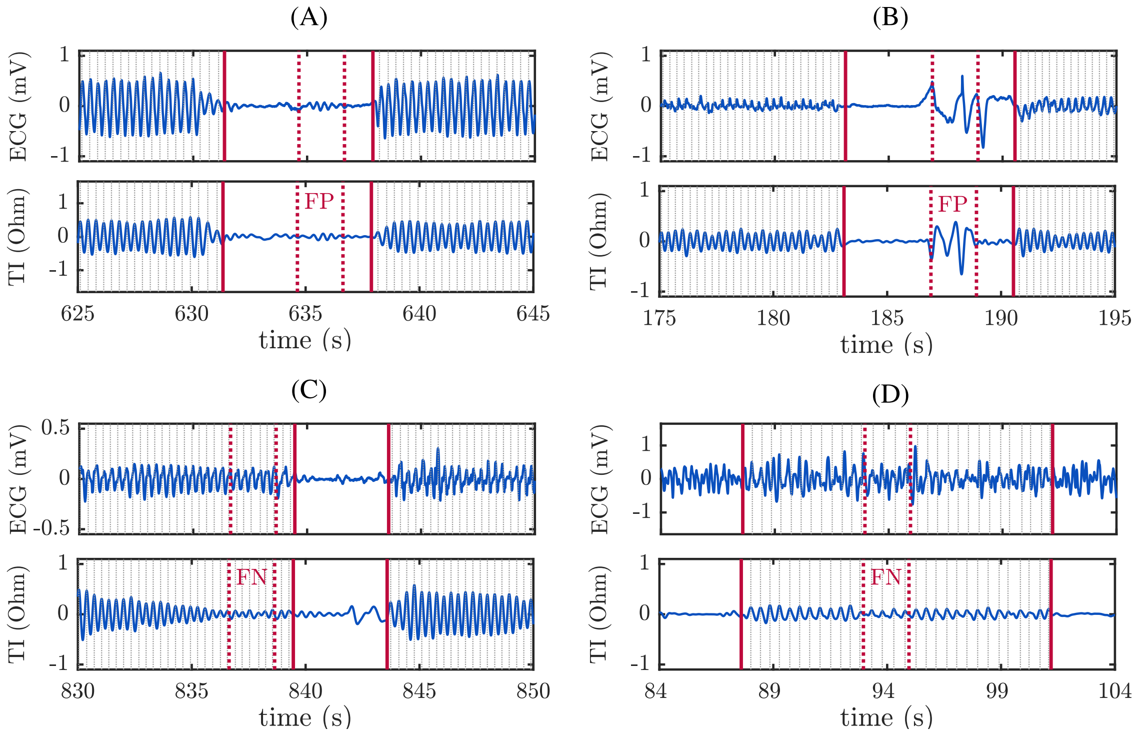

Fig 2. Examples of algorithm misclassification. (A, B) Compression activity detected during a compression pause. (C, D) Undetected compression activity in a chest compression series. Analyzed 2-s windows are delimited by red dotted lines, the beginning and end of chest compressions series with red solid lines. Grey dashed lines represent annotated individual chest compressions.
